# Metabolite derived from green tea polyphenol increases and activates plasmacytoid dendritic cells

**DOI:** 10.1007/s11418-025-01929-z

**Published:** 2025-07-05

**Authors:** Motofumi Kumazoe, Misato Nakajima, Reno Kawamoto, Yoshinori Fujimura, Reno Tomioka, Moeto Suzuki, Yuko Tanaka, Hirofumi Tachibana

**Affiliations:** 1 Division of Applied Biological Chemistry, Department of Bioscience and Biotechnology, Faculty of Agriculture, Kyushu University, 744 Motooka, Nishi-ku, Fukuoka, 819-0395 Japan; 2 R&D Unit, Mitsui Norin Company, Limited, 223-1 Miyabara, Fujieda-shi, Shizuoka 426-0133 Japan

**Keywords:** Green tea, Metabolites, Dendritic cells, Plasmacytoid dendritic cells, Immunity

## Abstract

**Abstract:**

The immune system plays a crucial role in protecting the body from harmful bacterial, viral infections and vital for cancer suppression. Dendritic cells (DCs) are indispensable mediators that facilitate the connection between innate and acquired immunity via antigen presentation and cytokine production. One of the major intestinal microbial metabolites of green tea polyphenols, 5-(3′,5′-dihydroxyphenyl)-γ-valerolactone (EGC-M5), enhances T cell activity. However, the detailed underlying mechanisms remain unknown. Here, we revealed that the oral administration of EGC-M5 increases and activates plasma cytoid dendritic cells (pDCs) in the spleen without causing changes in body weight. Consistent with these findings, administration of EGC-M5 increased the gene expression of interleukin-12 in the spleen. Oral administration of EGC-M5 significantly increased type I Interferon (IFN) and IL-6 levels, which are involved in vaccine-induced antibody production. Ex vivo experiments showed that EGC-M5 treatment did not directly enhance pDC differentiation. In conclusion, EGC-M5 indirectly increased pDC levels in vivo*,* accompanied by an increase in the expression of pDC activation markers and the gene expression of interleukin-12, type I IFN and IL-6 in the spleen.

**Graphical abstract:**

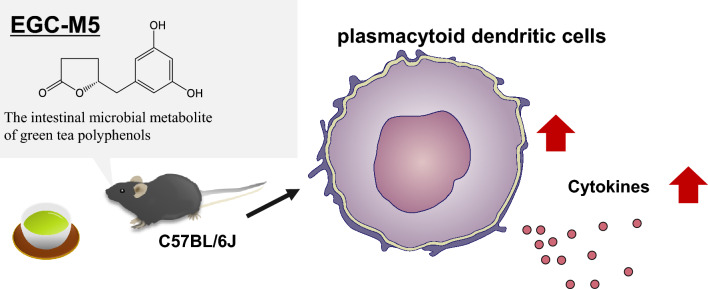

**Supplementary Information:**

The online version contains supplementary material available at 10.1007/s11418-025-01929-z.

## Introduction

The immune system is crucial for protection against various types of pathogens, including bacteria, viruses, and parasites [1]. The immune system consists of innate and acquired immunity, each with complex mechanisms that enhance and suppress each other [2, 3]. In addition, the activation of innate immunity is crucial for the appropriate elicitation of acquired immunity [3].

Dendritic cells (DCs) play an indispensable role as the interface between innate and acquired immunity [4]. DCs are the important professional antigen-presenting cells [4]. The expression of MHC class II acts as a checkpoint for this process by inducing the interactions between MHC class II and T cell receptors [5].

Plasmacytoid dendritic cells (pDCs) are a characteristic subset of dendritic cells that secrete high levels of type I interferon [6]. Recent studies have indicated a crucial role of pDCs in antiviral immune defense [7]. pDCs have a characteristic ability to secrete high levels of type I interferon, including IFN-α, in response to viral infection [8].

Moreover, the suppression of pDC function may induce chronic viral infection by suppressing T cell priming and viral clearance [9]. In antiviral defense, pDCs act as professional antigen-presenting cells via MHC class II molecules secrete high levels of type I interferon, and promote the cytotoxic activation of CD8^+^ T cells (cytotoxic T cells; CTLs) [7, 10] and NK cells by releasing cytokines including IL-12 [11].

Green tea, an extract of *Camellia sinensis* is one of the most widely consumed beverages in the world. A recent study showed that green tea intake is associated with longevity [12]. In addition, the systematic review and meta-analysis found that green tea catechins demonstrated statistically significant effects on the prevention of influenza infection compared with the control group (risk ratio (RR) 0.67, 95% CIs 0.51–0.89, P = 0.005) [13]. On the other hand, it has been shown that the amount of catechins absorbed into the body is very low and that most orally ingested catechins are broken down in the intestine by the action of intestinal bacteria before being absorbed into the body [14].

Therefore, we have focused on 5-(3′,5′-dihydroxyphenyl)-γ-valerolactone (EGC-M5), one of the major green tea polyphenol metabolites, which is produced by intestinal bacteria from epigallocatechin gallate (EGCG), the major catechin in green tea [15], and investigated its physiological activities on the immune system. Previously, we showed that EGC-M5 increases the activity of CD4^+^ T cells, and also enhances the cytotoxicity of natural killer (NK) cells in vivo [16]. However, the mechanism of these events is still unknown.

In this report, we revealed the effect of EGC-M5, a characteristic metabolite derived from green tea catechin, on the activity of dendritic cells, which play an indispensable role as the interface between innate and acquired immunity.

## Materials and methods

### Reagents

We obtained EGC-M5 through microbial cultivation as previously described [17]. The purity of EGC-M5 was confirmed by high-performance liquid chromatography (HPLC) analysis (at least 95%). Roswell Park Memorial Institute (RPMI) 1640 medium was purchased from Fujifilm Wako Pure Chemical Co., Ltd. (Osaka, Japan). Fetal bovine serum (FBS) was purchased from Sigma-Aldrich (St. Louis, MO, USA). 2-mercaptoethanol and 4% paraformaldehyde (PFA) were purchased from Fujifilm Wako Pure Chemical Co. Ltd. (Osaka, Japan). GM-CSF was obtained from Fujifilm Wako Pure Chemical Co. Ltd. (Osaka, Japan). LPS was purchased from Sigma-Aldrich (St. Louis, MO). Streptomycin and Penicillin G were purchased from Meiji Pharmaceutical Co. (Tokyo, Japan).

### In vivo experiment

All animal studies were conducted in compliance with the Law of Act on Welfare and Management of Animals (No. 105), the Standards Relating to the Care and Keeping and Reducing Pain of Laboratory Animals (Notice of the Ministry of the Environment No.88 of 2006), Fundamental Guidelines for Proper Conduct of Animal Experiment and Related Activities in Academic Research Institutions under the jurisdiction (Notice of the ministry of Education, Culture, Sports, Science and Technology No. 71 of 2006), the Kyushu University Animal Experiment Regulations and Administrative Instructions Kyushu University Animal Experiment Regulations. The animal experiments were approved by the Kyushu University Animal Care and Use Committee in Fukuoka, Japan (A23-127–1). Six-week-old C57BL/6J male mice, purchased from Kyudo (Tosu, Saga), were housed in a room with controlled humidity (50–70%) and temperature (25 ± 2 °C) on a 12 h dark–light cycle (light from 8:00 to 20:00). They had access to fresh autoclaved water and MF diet (KBT Oriental, Tokyo, Japan) ad libitum*.* After 1 week of acclimation, the mice were randomly divided into three groups: Gp1 mice were treated with vehicle (2.5% DMSO in injection water) (n = 8), Gp2 mice were treated with EGC-M5 30 mg/kg b.w. (2.5% DMSO in injection water) orally for 12 days, and Gp3 mice were treated with EGC-M5 100 mg/kg b.w. (2.5% DMSO in injection water) orally for 12 days. On the final day, mice were sacrificed under an isoflurane atmosphere. Organs, including the blood, spleen, and femur, were harvested and analyzed.

### Antibodies and flow cytometry

Antibodies for flow cytometry were purchased from BioLegend (San Diego, California, United States). Detailed information is provided in SI Table 1. All antibodies were diluted with 0.1% sodium azide and 2.5% bovine serum albumin (BSA) PBS. Cytometry was performed using the Verse (BD Biosciences). The harvested spleens were placed in a 5 mL dish containing RPMI-1640 medium, and the tissues were crushed using a glass slide to disperse the cells. The tissue fragments were removed by filtering through a mesh, followed by centrifugation for 5 min.

After removing the supernatant, the cells were treated with BD Pharm Lyse™ Lysing Buffer (Becton, Dickinson and Company), which was diluted 10 times with Otsuka distilled water (Otsuka Pharmaceutical). The cells were washed twice with RPMI-1640 medium and the cell count was measured using a hemocytometer. The cell concentration was adjusted to 1 × 10^7^ cells/mL. The cells were fixed in 2% formaldehyde solution for 20 min at 4 °C, centrifuged to remove the supernatant, then incubated at room temperature for 30 min in 5% FBS-TPBS (PBS containing 0.05%Tween-20) to permeabilize the cell membranes. The cells were then stained with 5% FBS-TPBS and analyzed using a flow cytometer, Verse™ (Becton, Dickinson and Company). Plasmacytoid dendritic cells (pDCs) were identified as CD11c^int^ CD3^–^ B220^+^cells, conventional dendritic cell subset 1 (cDC1) as CD11c^+^ CD3^–^ CD8^+^ cells, and conventional dendritic cell subset 2 (cDC2) as CD11c^+^ CD3^–^ CD11b^+^ cells.

The femur was placed in a 5 mL dish containing RPMI1640 medium, and the bone marrow was collected using a 2.5 mL syringe and a 26G needle. Tissue fragments were removed by mesh filtration. The supernatant was discarded and the cells were treated with BD Pharm Lyse™ Lysing Buffer. The cells were then washed twice with RPMI1640 medium and counted using a hemocytometer. The cells were fixed in 2% formaldehyde solution for 20 min at 4 °C, left to stand for 30 min at room temperature in 5% FBS-TPBS, and then permeabilized. Samples with obviously abnormal gate conditions were excluded from the analysis.

### Ex vivo study

The femur was placed in a 6 well plate containing RPMI1640 medium, and the bone marrow was collected using a 2.5 mL syringe and a 26G needle. Tissue fragments were removed by mesh filtration. Myeloid cells were seeded with or without EGC-M5 in 10% FBS RPMI1640 medium supplemented with Flt-3L (50 ng/mL and 50 μM 2-mercaptoethanol) at a density of 5 × 10^6^ cells/mL. After 5 days of incubation, the medium was removed and the cells were treated with fresh medium containing the same supplements. After 48 h of incubation, the pDC population was assessed using flow cytometry.

### qRTPCR

The harvested spleens were homogenized in 700 µL of TRI Reagent (Cosmo Bio, Tokyo, Japan) and added with 200 µL of chloroform (Nacalai tesque, Kyoto, Japan). After centrifugation (12,000×*g* for 15 min at 4 °C), 300 µL of the aqueous phase was collected and 300 µL of 2-propanol was added and mixed. After further centrifugation at 4 °C at 12,000×*g* for 10 min, the supernatant was removed and the pellet was washed with 700 µL of 75% ethanol. After another centrifugation (12,000×*g* for 15 min at 4 °C), the supernatant was removed, and the RNA pellet was dried. The pellet was dissolved in Nuclease-Free Water (NFW) (Ambion, TX, U.S.A.), and the RNA concentration was measured using a NanoDrop 2000 (Thermo Scientific, Kanagawa, Japan). cDNA was synthesized using a Prime Script™ RT Reagent Kit (Takara Bio, Shiga, Japan) in a thermocycler (Astec, Fukuoka, Japan). A volume of 1 µL cDNA was mixed with 2.5 µL of Sso AdvancedTM Universal SYBR^®^ Green Supermix (BIO-RAD, California, U.S.A.), 0.2 µL each of primers F and R, and 1.1 µL of ultra-pure water, and gene expression was analyzed using the CFX96TM Real-Time PCR Analysis System (BIO-RAD, California, U.S.A.). The denaturation temperature was set at 95 °C, the annealing temperature at 60 °C, and the amplification was performed for 50 cycles. The primer sequences are listed below (Table 1).

**Table 1:** Primers

Il12a	Forward; 5′-TCTGGTACATCTTCAAGTCCTCATAGA-3′
	Reverse; 5′-TACTAGAGAGACTTCTTCCACAACAAGAG-3′
Il12b	Forward; 5′-AACTTGAGGGAGAAGTAGGAATGG-3′
	Reverse; 5′-GGAAGCACGGCAGCAGAATA-3′
Ifng	Forward; 5′-GTATTGCCAAGTTTGAGGTCAAC-3′
	Reverse; 5′-GCTTCCTGAGGCTGGATTC-3′
Tnf	Forward; 5′-GAGGCACTCCCCCAAAAGAT-3′
	Reverse; 5′-CGATCACCCCGAAGTTCAGT-3′
Ifna	Forward; 5′-GTGACCTTCCTCAGACTCATAAC-3′
	Reverse; 5′-CAAAGTCCTTCCTGTCCTTCA-3′
Ifnb1	Forward; 5′-CCTGGAGCAGCTGAATGGAA-3′
	Reverse; 5′-CCACCCAGTGCTGGAGAAAT-3′
Il6	Forward; 5′-TCACAGAAGGAGTGGCTAAGGACC-3′
	Reverse; 5′-ACGCACTAGGTTTGCCGAGTAGAT-3′
Il10	Forward; 5′-CAGCCGGGAAGACAATAACTG-3′
	Reverse; 5′-CCGCAGCTCTAGGAGCATGT-3′
Rsad2	Forward; 5′-CTTCAACGTGGACGAAGACA-3′
	Reverse; 5′-GACGCTCCAAGAATGTTTCA-3′
Batf3	Forward; 5′-AGAAGGCTGACAAGCTCCAC-3′
	Reverse: 5′-GTGCACGAAGTGTTGCTTTG-3′
Xcr1	Forward; 5′-ACATGATACCCATGGGGAAGT-3′
	Reverse: 5′-GTGCACGAAGTGTTGCTTTG-3′
Siglech	Forward; 5′-ACTAAGCTTAGACAGGAGCCCAGGCCATC-3′
	Reverse: 5′-ACTGGATCCACTTACCTGTGGTGACATTGAGCTGGATAG-3′

### Statistical analysis

All results are shown as the mean ± standard error (SE). Significant differences were assessed using Student’s t-test and Dunnett’s test using GraphPad 8.0 (GraphPad Software). Statistical significance was set at *P* < 0.05. Outliers were identified using the ROUT test (Q = 1%).

## Results

### EGC-M5 treatment increased pDC population and cytokine expression levels in vivo

**Fig. 1:**
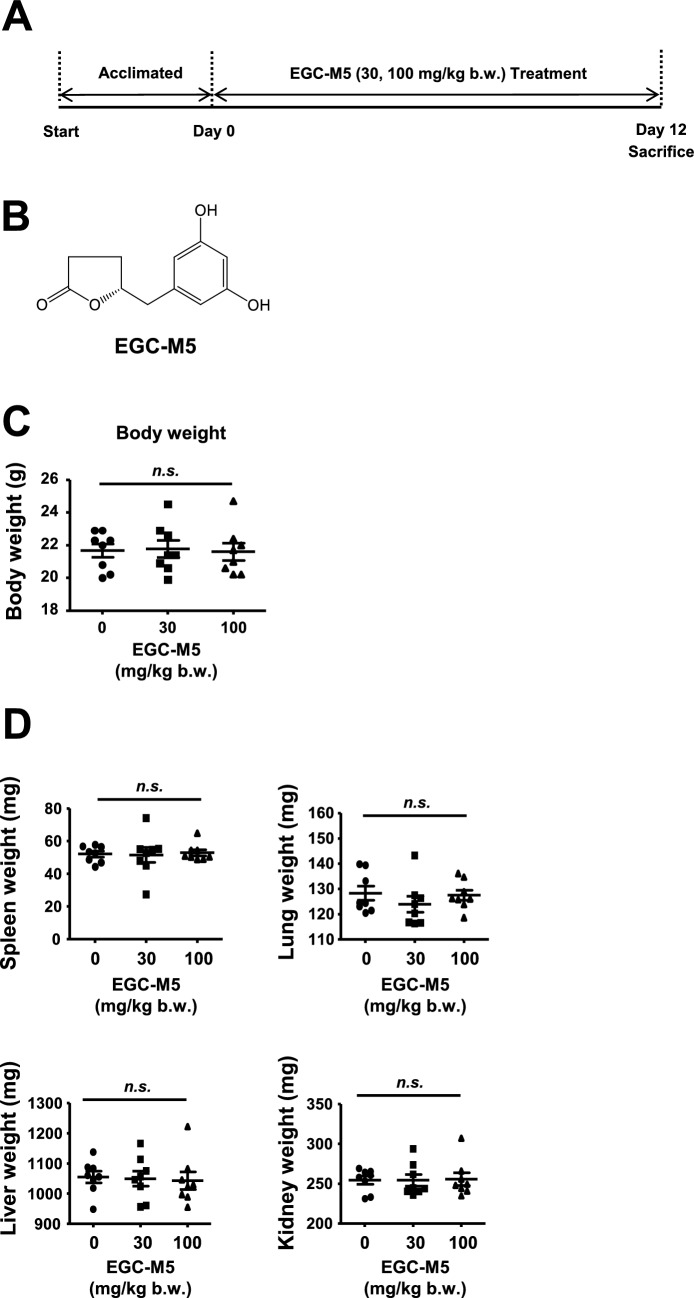
The effect of oral intake of EGC-M5 on bodyweight. **A** Experiment scheme. **B** Chemical structure of EGC-M5. **C**, **D** Six-week-old male C57BL/6J mice were administered with EGC-M5 for 12 days and the effect of EGC-M5 on the mice body weight and organ weight were measured. Mean ± S.E., n = 8, Dunnett’s test

We have previously reported that EGC-M5 treatment increases the activity of T cells [16]. DCs play a crucial role in regulating T cell activation [18]; therefore, we hypothesized that EGC-M5 upregulates T cell activity by activating dendritic cells. To assess the effects of EGC-M5, 6-week-old male C57BL/6J mice were randomly divided into three groups (control, low-dose EGC-M5, and high-dose EGC-M5) and orally administered EGC-M5 (30 or 100 mg/kg b.w.) for 12 days (Fig. 1A, B). EGC-M5 treatment did not affect the body weight of the mice (Fig. 1C) or the weight of organs, including the spleen, lungs, liver, and kidneys (Fig. 1D).

**Fig. 2:**
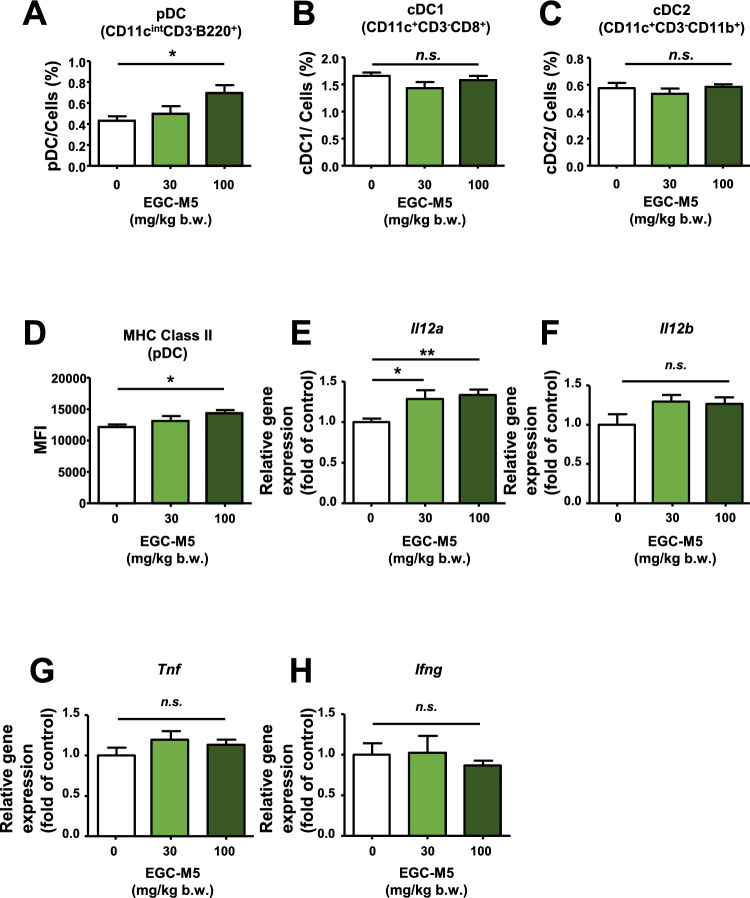
Oral intake of EGC-M5 increased the pDC population and enhanced pDC activation. **A**–**D** Six-week-old male C57BL/6J mice were administered with EGC-M5 for 12 days and spleens were harvested and the effect of EGC-M5 on the mice immune cell population and MHC class II expression of pDCs were evaluated by flow cytometry analysis. Plasmacytoid dendritic cells (pDCs) were identified as CD11c^int^ CD3^–^ B220^+^cells, conventional dendritic cell subset 1 (cDC1) as CD11c^+^ CD3^–^ CD8^+^ cells, and conventional dendritic cell subset 2 (cDC2) as CD11c^+^ CD3^–^ CD11b^+^ cells. **E**–**H** mRNA levels of cytokines were measured using qRTPCR. Mean ± S.E., n = 7–8, Dunnett’s test

To assess the effect of oral intake of EGC-M5, mouse spleens were harvested, and pDCs were evaluated by flow cytometry analysis. Our results showed that the oral intake of EGC-M5 significantly upregulated pDC levels in the spleen (Fig. 2A). In contrast, conventional dendritic cell (cDC1 and cDC2) levels were not affected by EGC-M5 (Fig. 2B, C).

Activated DCs activate T cells. Major histocompatibility complex (MHC-II) molecules play a crucial role in this process [7, 10]. In this context, MHC-II serves as a marker of activated dendritic cells. Our results showed that oral intake of EGC-M5 increased MHC-II expression in pDCs (Fig. 2D).

Taken together, EGC-M5 treatment increased the pDC population and activated pDC levels in the spleen.

Cytokines play a crucial role in regulating the immune system, including T cell activity [7, 10]. pDCs produce several types of cytokines, and pDC-induced T cell activation induces the expression of various cytokines [7, 10, 11]. These cytokines play crucial roles in regulating acquired immunity [7, 10, 11].

Interleukin-12 (IL-12) is a major Th1 cytokine that plays an essential role in cytotoxic T cell activation by differentiating naïve T cells into Th1 cells [18, 19]. In addition, IL-12 upregulates NK cell activity and induces interferon gamma production [19]. Professional antigen-presenting cells, including DCs, are the major source of IL-12 [19].

**Fig. 3:**
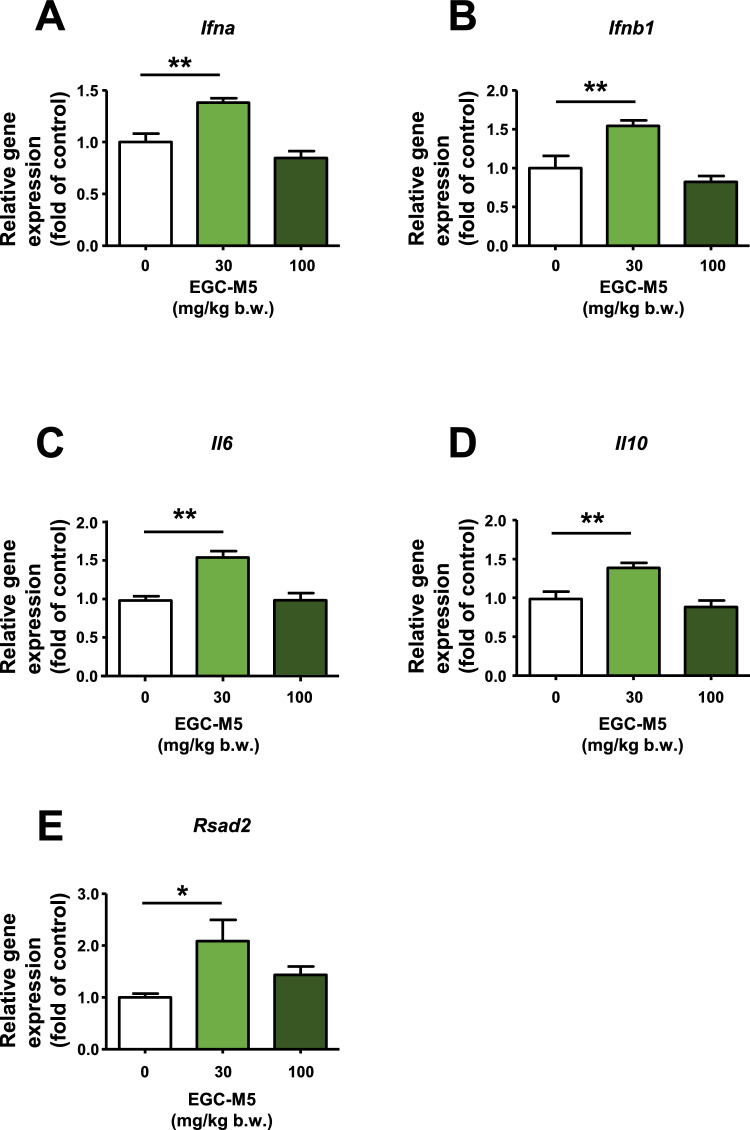
EGC-M5 treatment increased cytokine expression levels in the mice spleen. **A**–**E** Six-week-old male C57BL/6J mice were administered with EGC-M5 for 12 days and spleens were harvested and mRNA levels of cytokines were measured using qRTPCR. Mean ± S.E., n = 7–8, Dunnett’s test

Our results showed that oral EGC-M5 intake upregulated *Il12a* expression in the spleen in a dose-dependent manner (Fig. 2E). In contrast, it did not affect the expression of *Il12b*, tumor necrosis factor alpha (*Tnf*), and interferon gamma (*Ifng*) (Fig. 2F–H). Interestingly, 30 mg/kg EGC-M5 intake increased the gene expression of Interferon-alpha (*Ifna*), Interferon-beta1 (*Ifnb1*), Interleukin-6 (*Il6*), Interleukin-10 (*Il10*), and Radical *S*-Adenosyl Methionine Domain Containing 2 (*Rsad2*) in mouse spleen, which are crucial for dendritic cell maturation (Fig. 3A–E) [20]. Importantly, in pDCs, the upregulation of type I interferons plays a crucial role in antiviral defense. In contrast, 100 mg/kg EGC-M5 intake did not increase the expression of Ifnα, Ifnβ1, Il6, Il10, and Rsad2 in mouse spleen (Fig. 3A–E).

### EGC-M5 treatment affected the key transcription factors in DCs

**Fig. 4:**
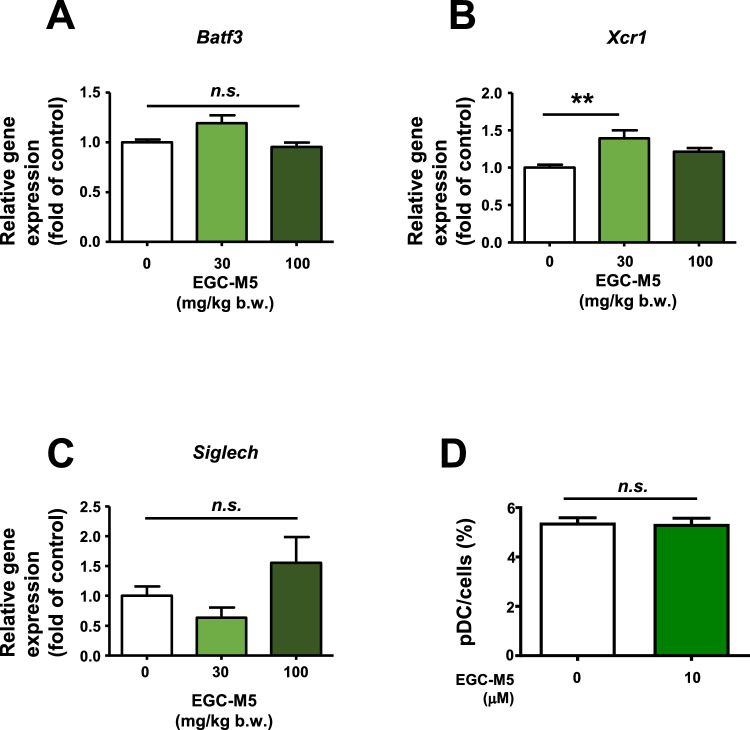
EGC-M5 treatment affected the expression of DCs-related genes. **A**–**C** Six-week-old male C57BL/6J mice were administered with EGC-M5 for 12 days and spleens were harvested and mRNA levels of DCs related genes were measured using qRTPCR. n = 7–8, Dunnett’s test. **D** Myeloid cells were seeded with or without EGC-M5 in medium supplemented with Flt-3L (50 ng/mL) and 50 μM 2-mercaptoethanol. After 5 days of incubation, the medium was removed and the cells were treated with fresh medium containing the same supplements. After 48 h of incubation, the pDC population was assessed using flow cytometry. Mean ± S.E., n = 4, Student’s *t*-test

Since DCs are the major source of type I interferons [7, 10], the effect of EGC-M5 intake on the expression levels of Basic Leucine Zipper ATF-Like Transcription Factor 3 (*Batf3*) was assessed because Batf3 plays a crucial role in DC function as a transcription factor [21]. Our results showed that 30 mg/kg EGC-M5 intake tended to increase the expression of *Batf3*; however, it was not statistically significant (Fig. 4A).

X-C motif chemokine receptor 1 (XCR1) is a receptor for lymphotactin-1/2 (X) [22] and is widely expressed in DCs. This receptor plays a crucial role in antigen cross-presentation. A recent study has shown that XCR1^+^ DCs are essential for antiviral defense. Interestingly, EGC-M5 30 mg/kg oral treatment significantly increased *Xcr1* gene expression in the spleen, whereas EGC-M5 100 mg/kg oral treatment did not affect its expression (Fig. 4B).

Siglec-H is a receptor protein that is characteristically expressed in pDCs and negatively regulates IFN-α production [23, 24]. Oral administration of EGC-M5 did not affect the expression levels of *Siglech* (Fig. 4C). As mentioned above, treatment with EGC-M5 increased pDC levels in the spleens of mice (Fig. 2A). Therefore, we assessed the effect of EGC-M5 on pDC differentiation. To evaluate this, since pDCs are derived from bone marrow cells [10], myeloid cells were harvested and cells were seeded with or without EGC-M5 in medium supplemented with Flt-3L (50 ng/mL) and 50 μM 2-mercaptoethanol. After 7 days of incubation, the pDC population was assessed using flow cytometry. Our results demonstrated that EGC-M5 treatment did not significantly increase pDC levels (Fig. 4D).

## Discussion

A clinical study has shown that green tea intake is negatively correlated with risk of influenza infection [13]. A case–control study in a cohort of 4302 workers found that the influenza risk for green tea consumption of ≥ 5 cups/week was 0.61 (95% CI 0.39–0.95) compared with < 1 cup/week [25]. In an epidemiological study, green tea consumption (1–5 cups/day) was inversely associated with the risk of influenza infection in children [26] (3–5 cups/day compared with < 1 cup/day were 0.54). However, little is known about the detailed mechanisms.

Catechins are recognized as the primary bioactive components of green tea. On the other hand, previous reports showed that intact catechins could not easily be abosorbed, and that the majority of catechins consumed orally are degraded in the intestines by intestinal bacteria prior to absorption into the body [14]. In addition, we previously reported that EGC-M5, a characteristic metabolite derived from green tea catechins, exhibits immune activation effects in T cells and NK cells [16]. However, the underlying mechanisms of these effects have not been fully elucidated. Dendritic cells, known for activating immune cells such as T cells and NK cells through antigen presentation and cytokine production, might play a role [7, 10]. Here, we showed that oral intake of EGC-M5 100 mg/kg increased pDC in the spleen with an increase in the expression of MHC-class II. Oral intake of EGC-M5 also increased the expression of *Il12a* in the spleen, a constituent subunit of cytokine IL-12. Additionally, EGC-M5 30 mg/kg induced IFN-I expression in the spleen. The production of IL-12 and IFN-I by pDCs is known to enhance NK cell activation and support both the accumulation and effector functions of CD8^+^ T cells, along with aiding the polarization of CD4^+^T cells [7, 10, 11]. Therefore, the observed activation of CD4^+^T cells and NK cells by EGC-M5 in the previous report [16] could potentially be attributed to the increased population and enhanced activation of pDCs.

Additionally, when bone marrow cells, including pDC progenitor cells, were cultured in pDC differentiation-inducing media with EGC-M5, no increase in pDC levels was observed compared to the untreated group. Thus, the enhancement of differentiation into pDCs is likely an indirect effect of EGC-M5 on dendritic precursor cells.

In this study, the increase in pDC levels and IFN-I gene expression were observed following EGC-M5 administration; however, their concentration dependency was not consistent. The increase in pDC population and cytokine expression may reflect distinct regulatory mechanisms or differences in the timing of the response. Moreover, since the directions of these effects appear to differ, it is likely that a clear dose-dependent relationship cannot be observed.

In addition, our results suggest that EGC-M5 modulates cytokine expression in splenic immune cells, but we could not definitively distinguish whether the observed effect was mediated by dendritic cells or macrophages, as both populations are present in low abundance. Therefore, it remains possible that the effect of EGC-M5 may involve macrophages as well. Further studies are needed to clarify the cell-type-specific mechanisms of EGC-M5 action.

To prevent influenza, clinically used split-virus vaccines include trivalent inactivated vaccines (TIV) and quadrivalent inactivated influenza vaccines (QIV). Importantly, split-virus vaccines are well tolerated in patients with modest effects (based on the meta-analysis, efficacy would be 60%) [27], and further improvement is strongly demanded. Oral administration of EGC-M5 at a low dose (30 mg/kg) significantly increased type I IFN and IL-6 levels, which are involved in vaccine-induced antibody production [11]. Additionally, in our study, the oral administration of EGC-M5 at a low dose (30 mg/kg) significantly increased *Xcr1* expression. XCR1 is involved in the T-cell-mediated immune response against chronic viral infections [22]; therefore, EGC-M5 may enhance the T-cell immune system. EGC-M5 could aid in the treatment and prevention of virus-assosiated diseases by enhancing the efficacy of the virus vaccine and T-cell-mediated immune response against viral infections.

Although no exogenous antigen was administered in this study, the immune system of mice is constantly exposed to endogenous and environmental antigens, such as gut microbial components. Thus, the observed enhancement of pDC activation by EGC-M5 is considered to reflect its modulatory effect under physiological conditions of continuous antigenic stimulation. Further studies using exogenous antigens are required to assess the effects of EGC-M5 on vaccine-induced antibody production and antiviral defense of the immune system.

In this study, Batf3 activation was assessed based on mRNA expression levels. However, mRNA expression does not necessarily reflect the functional activation state of the protein. Since Batf3 is known to be regulated by post-translational modifications such as phosphorylation, future studies should evaluate the phosphorylation levels of Batf3.

In conclusion, oral administration of EGC-M5 induced the upregulation of the pDC population and MHC class II expression in pDCs and increased the expression of several types of cytokines, including type I IFNs and IL-6. Thus, EGC-M5 and catechins that degrade to EGC-M5 are promising candidates for the therapy and prevention of virus-related diseases.

## Supplementary Information

Below is the link to the electronic supplementary material.

Supplementary file1 (DOCX 35 kb)

## Data Availability

The datasets generated and/or analyzed during the current study are not publicly available but are available from the corresponding author upon reasonable request. None of the participants was directly involved in this study.
