# Supplementary material for: Metabolite derived from green tea polyphenol increases and activates plasmacytoid dendritic cells
Source: J Nat Med. 2025 Jul 5;79(5):1057–66. doi: 10.1007/s11418-025-01929-z (PMC12408756; doi:10.1007/s11418-025-01929-z)
Supplement: Supplementary file 1 — Supplementary file1 (DOCX 35 kb) [file 11418_2025_1929_MOESM1_ESM.docx]

**SI Table. 1 FACS and antibodies**

| **Name** | **Company** | **Cat** |
| --- | --- | --- |
| BD FACS Flow | BD | 342003 |
| BD Pharm Lyse | BD | 555899 |
| BD FACSFlow | BD | 342003 |
| FITC anti-mouse CD3 | Biolegend | 100204 |
| PE anti-mouse CD11c Antibody | Biolegend | 117308 |
| PerCP/Cyanine5.5 anti-mouse/human CD11b Antibody | Biolegend | 101230 |
| APC anti-mouse I-A/I-E (MHC-II) | Biolegend | 107614 |
| PerCP/Cy5.5 amti-mouse CD103 Antibody | Biolegend | 121416 |
| Brilliant violet 510 anti-mouse/human CD45R/B220 Antibody | Biolegend | 103248 |
